# Morphological and Molecular Characterization of Human Gingival Tissue Overlying Multiple Oral Exostoses

**DOI:** 10.1155/2019/3231759

**Published:** 2019-05-22

**Authors:** Luca Francetti, Claudia Dellavia, Stefano Corbella, Nicolò Cavalli, Claudia Moscheni, Elena Canciani, Nicoletta Gagliano

**Affiliations:** ^1^Department of Biomedical, Surgical and Dental Sciences, Università degli Studi di Milano, via L. Mangiagalli 31, 20133 Milan, Italy; ^2^IRCCS Istituto Ortopedico Galeazzi, Via R. Galeazzi, 4, 20161 Milan, Italy; ^3^Department of Biomedical and Clinical Sciences “L. Sacco”, Università degli Studi di Milano, via G.B. Grassi 74, 20157 Milan, Italy; ^4^Department of Biomedical Sciences for Health, Università degli Studi di Milano, via L. Mangiagalli 31, 20133 Milan, Italy

## Abstract

Gingival and osseous augmentations are reported as hypertrophic or hyperplastic reactions to different factors including chronic traumatisms and surgeries such as free gingival graft (FGG) that induce an abnormal growth of both hard and soft tissues in genetically predisposed subjects. Since an imbalance in collagen turnover plays a key role in the development of gingival overgrowth leading to an accumulation of collagen in gingival connective tissue, in this study we described the histological and molecular features of three oral overgrowths obtained from a 34-year-old woman previously operated for FGG in order to evaluate a possible relationship between exostoses and overgrown tissue. Healthy and overgrown gingiva were analyzed by histological methods, and the expression of genes and proteins involved in collagen synthesis, maturation, and degradation was assessed in cultured fibroblasts obtained from gingival fragments at the molecular level. Our results show that general morphology and collagen content were similar in healthy and overgrown gingivae. However, fibroblasts obtained from the overgrown gingiva revealed an anabolic phenotype characterized by an increased collagen turnover and maturation. These findings indicate that an exostosis could act as a mechanical stimulus stretching the overlying connective tissue and triggering an anabolic phenotype of gingival fibroblasts and suggest to use minimally invasive surgical techniques to avoid traumatizing the periosteal tissues for the eradication of the exostosis with minimal relapses.

## 1. Introduction

Exostoses are described in literature as benign localized overgrowths of the bone of unknown etiology [1]. They may appear as nodular, flat, or pedunculate protuberances and may be found in different anatomical areas; the most frequent are palatal and mandibular *tori*, but vestibular exostoses can also be present. Vestibular exostoses are most common in the maxilla and are less frequently found on the mandibular surface. Multiple exostoses are present less regularly [1, 2]. Exostoses may appear as a rare side effect of free gingival graft (FGG) resulting from periosteal traumatism during site preparation that induces the activation of osteoprogenitor cells and of fibroblasts contained in the connective tissue of the graft, leading to bone and soft tissue overgrowth [1, 3].

Several studies have tried to explain the etiology, and different hypotheses have been proposed. Chambrone and Chambrone evaluated nine exostoses where FGG surgery was performed to augment the amount of keratinized gingiva. The authors have observed that in each of the examined cases, intentional or accidental periosteal fenestration during the surgical approach has occurred [1]. Considering the hypotheses of periosteal trauma acting as the main etiologic agent, frenectomies can also be involved in exostosis formation. In fact, the linear micro-fenestrations that occur during the preparation of a receiving site can evoke bone stimulation leading to the development of exostoses [2]. In three cases described in literature, the exostoses appeared in grafted areas associated with teeth that had supported removable or fixed prostheses. It is possible that the combination of periosteal fenestration and occlusal function as a continuous irritation factor is responsible for this osseous proliferation [4–6]. Pechenkina and coworkers in 2002 studied bone exostosis development in 66 well-preserved crania from Neolithic China to establish a possible etiopathological association [7]. The role of ethnic and genetic predispositions seemed to be important in the occurrence of such exostoses. In the same study, authors considered other factors as potentially related to exostosis formation such as masticatory hyperfunction (clenching or bruxism), occlusal stress, a compensatory response of periodontal disease, and chemical agents and concluded that every periodontal microdamage or inflammation can evolve in exostoses in genetically predisposed subjects. Severe occlusal stress, associated with temporomandibular joint disorders, was hypothesized to be a major environmental factor predisposing to exostosis development [7]. Echeverria and colleagues in 2001 presented a report about the formation of exostoses following FGG surgical procedure [8]. The authors postulated that surgical trauma combined with occlusal stress and genetic factors could cause the occurrence of exostoses. They also noticed that most of the post-FGG exostoses appeared in the premolar area, probably due to excessive chewing forces. The histological examination of hard and soft tissues of exostoses showed dense and mature lamellar bone surrounded by fibrous tissue with the presence of acanthosis, pseudoepitheliomatous hyperplasia, parakeratosis, and reactive fibrosis [8]. Likewise, other authors proposed the same potential etiological explanation, suggesting that the periodontal trauma and surgical damage of soft tissue (plastic surgery of the vestibule, gingival graft, and, notably, fenestration occurred in the periosteum) can evolve in buccal bony exostoses in genetically predisposed patients [3, 9–14].

Since the mechanisms leading to the overgrowth of gingival connective tissue overlying the exostosis are not completely known, the aim of this study was to characterize the gingival connective tissue in the case of multiple exostoses following FGG by histological and molecular analysis.

## 2. Case Presentation

### 2.1. Patient

**Figure 1:**
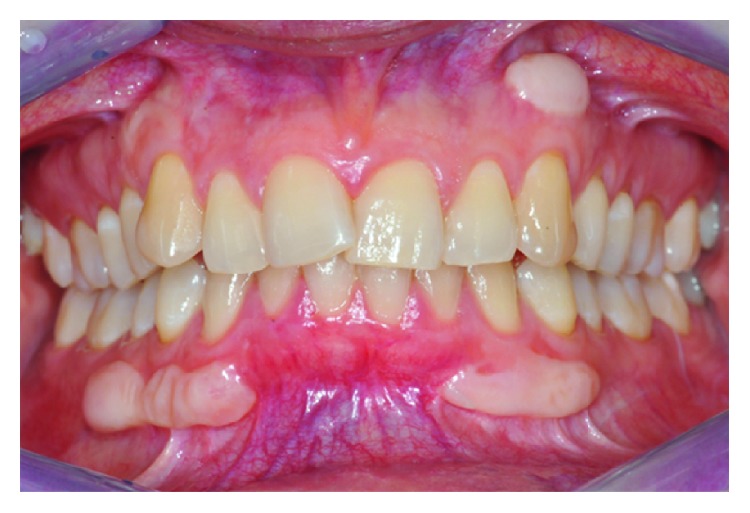
Clinical aspect at baseline.

A female patient, 34 years old at the time of the first visit, was referred to us to correct her gingival esthetic problem caused by the formation of gingival/mucosal overgrowths. Gingival outgrowths localized in the second, third, and fourth quadrants, corresponding to the position of premolars and canine roots, were identified during an oral examination. Their consistency appeared hard and unmovable, and they presented a normal gingival color. The lesions were asymptomatic, and the morphology did not change over time (Figure 1).

The anamnestic investigation revealed a history of FGG surgery in 2004 (8 years before our first examination) in each site that was treated for gingival recessions; allergic reactions to nonsteroidal anti-inflammatory drugs, paracetamol, cephalosporin, penicillin and its derivatives, and macrolide antibiotics; absence of any systemic conditions affecting metabolism; and altered wound healing (cutaneous keloids and hypertrophic scars).

The hypothesis of exostosis formation was suggested by the following clinical observations: benign aspect, nodular and hyperplastic tissue, hard and stable on palpation, not symptomatic, and localized in canine and premolar areas involved in a surgical procedure that could have acted as a promoter event.

We decided to perform an excisional surgery, followed by a histologic and molecular evaluation. The Review Board of the Research Center in Oral Implantology of the Università degli Studi di Milano in Milan, Italy, approved the treatment and research protocol before the first surgery.

All procedures were in accordance with the ethical standards of the institutional and/or national research committee and with the 1964 Helsinki declaration and its later amendments or comparable ethical standards. The patient was informed about the formulated medical hypothesis and the need for surgical intervention to remove the lesions and analyze the specimen. The patient agreed to the surgery and signed a written informed consent form. The surgery was planned after a complete evaluation of the patient's systemic conditions that confirmed an absence of clinical contraindications.

### 2.2. Surgical Procedures

All surgeries were performed in three different sessions by the same operator, with more than ten years of experience in the field of oral surgery (LF), and following the same protocol. The first surgical event involved a lesion localized in the second quadrant. Local anesthesia was performed with articaine 4% + epinephrine 1 : 100.000, voiding injection in the lesion area. The lesion was first isolated by incisions with a microblade following its external border; after that, the incision was continued above the periosteum, removing the tissue covering the exostosis. Then, with a 15C blade, a full-thickness incision was performed coronally to the lesion and the exostosis surface was exposed using a periosteal elevator. The full-thickness incision was extended in the mesiodistal direction in order to have a better view of the surgical site. Gingival fragments obtained from the overgrown gingiva were collected for histological and molecular characterization. A carbide bur, mounted on a rotary instrument and under sterile saline solution irrigation, was used to remove the bone structure in order to recreate a plane surface. To obtain a primary closure of the flap after the removal of the lesion, a partial thickness incision was performed in the free alveolar mucosa. The flap was then sutured with a 6/0 nonresorbable suture (ETHILON™ Nylon Suture, Ethicon ®, Cincinnati, OH, United States). After flap closure, an infiltration of 4 mg corticosteroid (BENTELAN 4 mg/2 mL, Defiante Farmaceutica S.A., Madeira, Portugal) was performed, to prevent the recurrence of overgrowths in the short term. The last surgical procedure was performed within 4 months of the first visit.

The follow-up visits revealed small relapses after 18 months from the surgical intervention that were asymptomatic and did not affect aesthetics and function. The recurrences observed in the second and fourth quadrants were linearly arranged along the incision of the periosteum that occurred during the surgery, while the lesion treated in the third quadrant showed a more evident relapse.

### 2.3. Histological Analysis

One gingival fragment was processed for histological analysis while another fragment was cultured for in vitro evaluations. Moreover, to characterize the pathological gingiva, a fragment from the healthy papilla was obtained from the same patient for comparison.

Immediately after surgery, each tissue fragment was fixed in 4% formalin in 0.1 M phosphate-buffered saline (PBS), pH 7.4, routinely dehydrated and paraffin-embedded. Serial sections of 5 *μ*m were obtained. Sections were stained with freshly made hematoxylin-eosin (Sigma-Aldrich, St Louis, MO, United States) to evaluate cell and tissue morphology.

To evaluate fibrillary collagen, slides were deparaffinized and immersed for 30 minutes in saturated aqueous picric acid containing 0.1% Sirius Red F3BA (Sigma-Aldrich, St. Louis, MO, United States), a staining method that specifically marks collagen [15]. The sections were observed under a light and polarized microscope (Nikon Eclipse E600, Nikon, Tokyo, Japan) and photographed with a calibrated digital camera (DXM1200, Nikon, Tokyo, Japan) at a 20x magnification.

### 2.4. Cell Cultures

Human gingival fibroblasts were obtained from a gingival fragment covering the exostosis and from a gingival fragment obtained from the patient's healthy gingiva. Tissue fragments were washed with sterile PBS, plated in T25 flasks, and incubated in DMEM containing 10% heat-inactivated fetal bovine serum (FBS) and antibiotics (100 U/mL penicillin, 0.1 mg/mL streptomycin) at 37°C in a humidified atmosphere with 5% CO_2_. Fibroblasts that grew out from the explant were trypsinized (0.025% trypsin-0.01% EDTA) for secondary cultures and plated in T75 flasks, as previously described [16, 17]. Human gingival fibroblasts were used between the fourth and fifth passages. Molecular evaluations were performed in samples cultured for 24, 48, and 72 h. All evaluations were performed on duplicate cultures for each sample.

### 2.5. Cell Morphology and Viability

Cell morphology of cultured gingival fibroblasts was analyzed by phase-contrast microscopy using a Leica DM IL microscope. Cell viability was determined by Trypan blue exclusion. Growth curves were obtained after plating cells in 6-well plates (150,000 cells/well). After the attachment, the cells were counted after 24, 48, and 72 h.

### 2.6. Real-Time RT-PCR

Total RNA was isolated from cultured fibroblasts as previously described [17]. Briefly, 1 *μ*g of total RNA was reverse-transcribed in a 20 *μ*L final volume of reaction mix (Bio-Rad, Segrate, Milan, Italy). Gene expression for the tissue inhibitor of matrix metalloproteinase 1 (TIMP-1), lysyl oxidase (LOX), and focal adhesion kinase (FAK) was assessed. GAPDH was used as internal control for normalization of the differences in the amount of total RNA in each sample. The following primers were used: GAPDH: sense CCCTTCATTGACCTCAACTACATG, antisense TGGGATTTCCATTGATGACAAGC, antisense GGAACCAGGATGACCAGATGTACC; TIMP-1: sense GGCTTCTGGCATCCTGTTGTTG, antisense AAGGTGGTCTGGTTGACTTCTGG; LOX: sense GGATACGGCACTGGCTACTT, antisense GACGCCTGGATGTAGTAGGG; and FAK: sense GTCTGCCTTCGCTTCACG, antisense GAATTTGTAACTGGAAGATGCAAG.

Amplification was performed in a 96-well plate in a final volume of 20 *μ*L per well containing 10 *μ*L of 1x SYBR Green Supermix (Bio-Rad, Italy), 2 *μ*L of template, and 300 pmol of each primer, and each sample was analyzed in triplicate in an iQ5 thermal cycler (Bio-Rad, Italy) after 40 cycles. After determining the cycle threshold (Ct), gene expression levels relative to that of GAPDH were calculated by the 2^ΔCt^ method.

### 2.7. Slot Blot

Slot blot analysis was used to assess type I collagen (COL-I), type III collagen (COL-III), and matrix metalloproteinase-1 (MMP-1) protein levels secreted by gingival fibroblasts in cell culture supernatants, as previously described [17]. Briefly, protein content in cell culture media was determined by a colorimetric assay (DC Protein Assay, Bio-Rad, Italy); 100 *μ*g of total proteins per sample (final volume of 200 *μ*L of Tris-buffered saline (TBS)) was spotted onto a nitrocellulose membrane using a Bio-Dot SF apparatus (Bio-Rad, Italy). After blocking for 1 h with 5% skimmed milk in TBST (TBS containing 0.05% Tween-20), pH 8, membranes were incubated for 1 h at room temperature in a monoclonal antibody to COL-I (1:1000 in TBST) (Sigma, Italy), to COL-III (1:2000 in TBST) (Sigma, Italy) and to MMP-1 (1 *μ*g/ml in TBST, Millipore). After washing with TBST, membranes were incubated in HRP-conjugated rabbit anti-mouse serum for 1 h (1 : 6000 in TBST to detect COL-I and COL-III or 1 : 40,000 in TBST to detect MMP-1, respectively) (Sigma, Italy). Immunoreactive bands were revealed by the Opti-4CN substrate or Amplified Opti-4CN substrate (Bio-Rad, Italy) and quantified by densitometric scanning (UVBand, Eppendorf, Italy).

### 2.8. SDS-Zymography

Serum-free cell culture media were mixed 3 : 1 with sample buffer (containing 10% SDS), as previously described [16, 17]. Five *μ*g of total protein per sample was run under nonreducing conditions without heat denaturation on 10% polyacrylamide gel (SDS-PAGE) copolymerized with 1 mg/mL of type I gelatin at 4°C. After SDS-PAGE, the gels were washed twice in a renaturing buffer (2.5% Triton X-100, Tris-HCl 50 mM) for 30 min each and incubated overnight in a substrate buffer at 37°C (Tris-HCl 50 mM, CaCl_2_ 5 mM, NaN_3_ 0.02%, pH 7.5). After staining the gels with Coomassie Brilliant Blue R-250, MMP gelatinolytic activity was detected as clear bands on a blue background. Bands were quantified by densitometric scanning (UVBand, Eppendorf, Italy).

### 2.9. Statistical Analysis

Data, expressed by mean ± standard deviation (SD), were analyzed by t-test. *p* values less than 0.05 were considered significant.

## 3. Results

### 3.1. Clinical Outcomes

**Figure 2:**
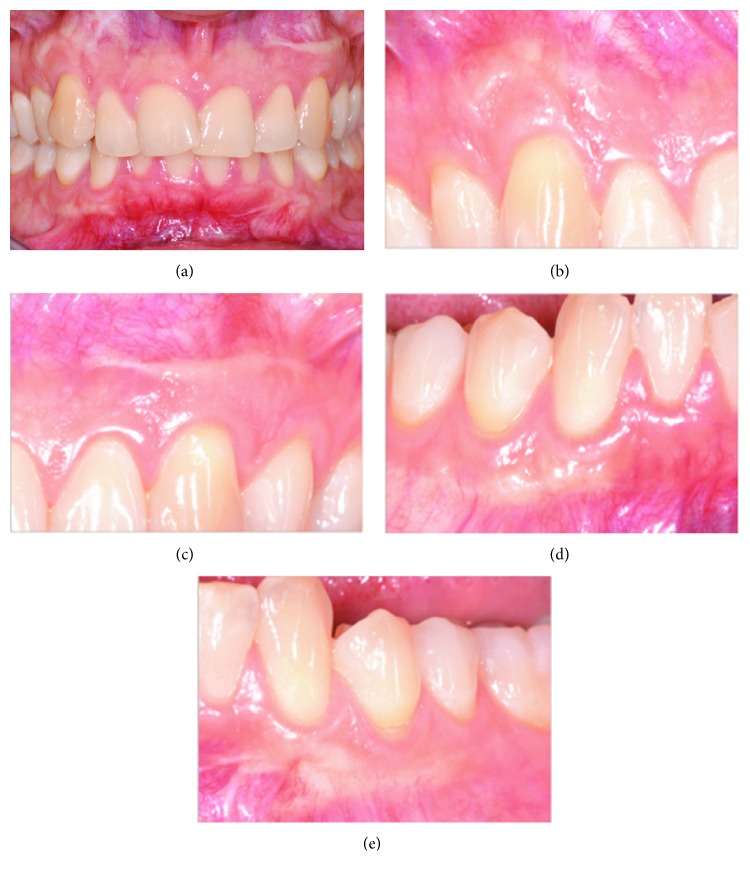
Clinical situation 4 years after the last surgical intervention. In the absence of gingival overgrowth, formation of thin scars is visible.

From a clinical point of view, in the last follow-up visit, after 4 years from the last surgical intervention, no exostosis or gingival overgrowth formations could be observed (Figure 2) and we recorded a high degree of satisfaction with the treatment by the patient. However, scars, where surgeries were performed, were visible and clearly distinguishable from the surrounding tissues. Palpation did not reveal any overgrowth of soft or hard tissue.

### 3.2. Histological Analysis

**Figure 3:**
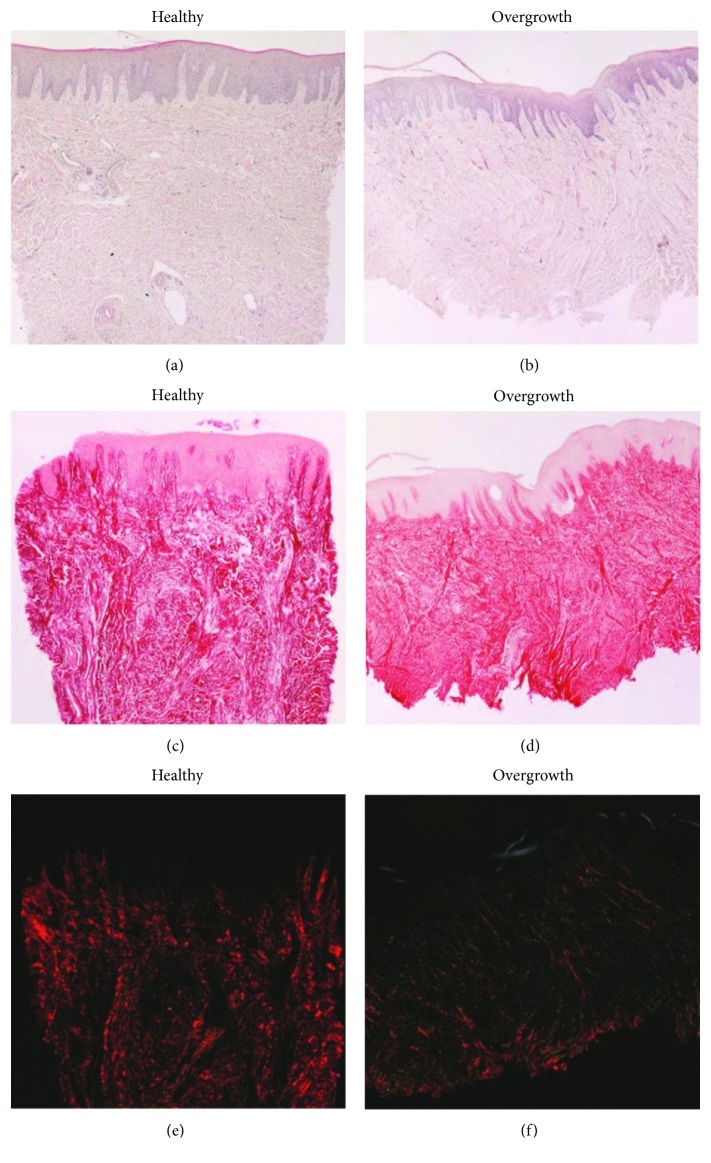
Micrographs showing the healthy and overgrown gingivae. (a-b) Hematoxylin and eosin staining reveals the morphological structure of the epithelium and of the connective tissue. (c-d) Sirius Red staining stains in red the collagen fibers of the connective tissue. (e-f) Polarized light performed on Sirius Red-stained samples shows birefringence relative to collagen fiber arrangement and maturation. Original magnification: 20x.

Light microscopy analysis of hematoxylin and eosin-stained samples revealed that tissue integrity and structure were maintained in healthy and overgrown tissues (Figures 3(a) and 3(b)). The lining squamous-stratified epithelium was orthokeratinized, displaying a normal structure and normal thickness of the *stratum corneum* in both healthy and overgrown tissues. The dense irregular connective tissue was characterized by well-organized collagen fibers and an extracellular matrix with blood vessels normally distributed and without any inflammatory infiltrates. Sirius red staining specifically stained collagen fibers, densely packed in an irregular array, and revealed that the healthy and overgrown gingivae displayed a similar collagen content and arrangement (Figures 3(c) and 3(d)). The presence of connective papillary projections throughout the epithelium was also evident. The analysis of Sirius Red-stained sections under polarized light allowed detecting some qualitative differences in the epithelium and in the lamina propria of the healthy and overgrown gingivae. The healthy epithelium was totally lacking birefringence, while the overgrown gingiva showed a thin and not uniform birefringent layer in the superficial portion. The connective tissue was also analyzed under polarized light. According to Junqueira et al., under polarized light, the collagen matrix is stained from green to yellow to orange-red depending on the orientation of the collagen bundles and the maturation of collagen [15]. Specifically, the newly secreted and less mature collagen is stained in green while the mature collagen is stained in orange-red. Healthy gingiva appeared red under polarized light (Figure 3(e)). By contrast, red birefringence was less evident in the overgrown gingiva and the connective tissue was stained green to yellow, suggesting that the collagen was less mature (Figure 3(f)).

### 3.3. Cell Viability

**Figure 4:**
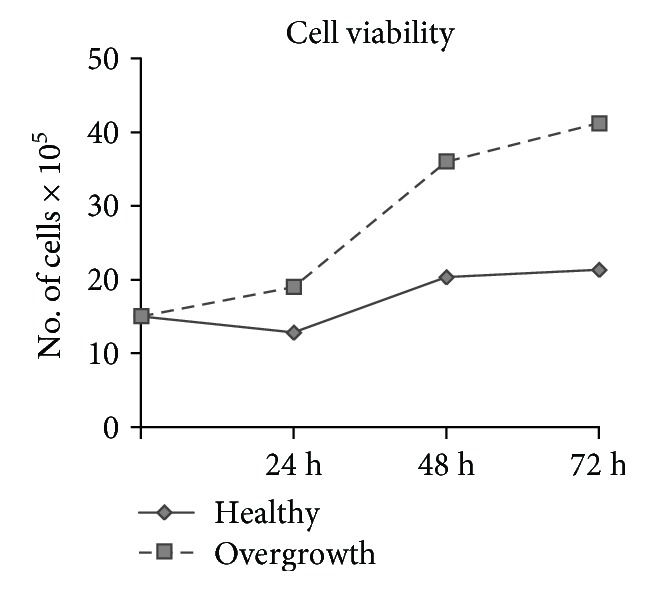
Cell proliferation. Growth curves showing cell viability in fibroblasts from healthy and overgrown gingivae after 24, 48, and 72 h.

Growth curves have shown that fibroblasts from the healthy and overgrowth gingivae exhibited a different proliferation rate at the considered time points. Fibroblasts from the overgrown gingiva tended to increase proliferation after 24, 48, and 72 h (Figure 4).

### 3.4. Collagen Turnover

**Figure 5:**
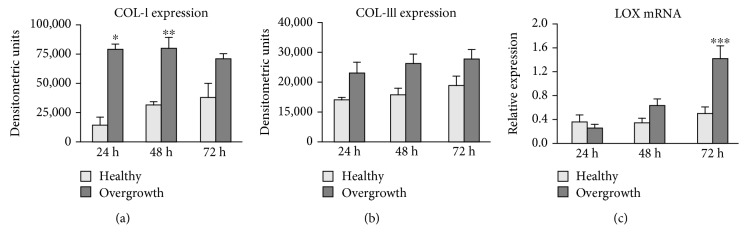
Collagen expression and maturation. Bar graphs showing COL-I (a) and COL-III (b) protein levels assessed by slot blot in cell culture supernatants of fibroblasts from healthy and overgrown gingivae. (c) Bar graphs showing LOX mRNA levels in fibroblasts from healthy and overgrown gingivae. GAPDH gene expression was used as internal control for normalization. Data are mean ± SD for samples run in duplicate. ^∗^*p* < 0.01, ^∗∗^*p* < 0.05, and ^∗∗∗^*p* < 0.005.

The histological findings were supported by the analysis of collagen turnover. Collagen content in gingival fibroblast supernatants revealed that fibroblasts from the overgrown gingiva synthesized and secreted higher COL-I (*p* < 0, 01, *p* < 0, 05, and *p* = 0,066 vs. healthy gingiva after 24, 48, and 72 h, respectively) and COL-III protein levels (*p* = 0, 08, *p* = 0, 09, and *p* ns vs. healthy gingiva after 24, 48, and 72 h, respectively) (Figures 5(a) and 5(b)).

Gene expression for LOX resulted to be strongly upregulated in fibroblasts from the overgrown gingiva (*p* < 0.005 vs. healthy at 72 h) (Figure 5(c)).

**Figure 6:**
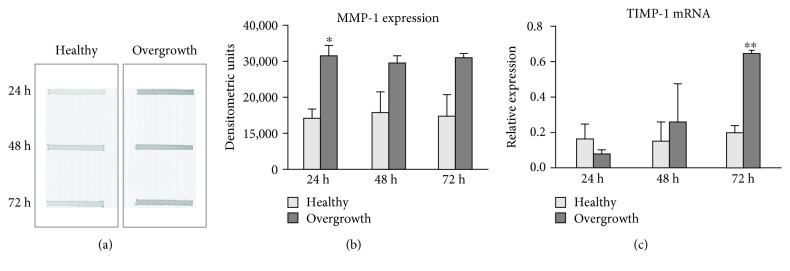
MMP-1 and TIMP-1 expression. (a) Representative slot blot showing MMP-1 levels in serum-free cell culture supernatants of fibroblasts from healthy and overgrown gingivae. Immunoreactive bands were quantified by densitometric scanning as described in Materials and Methods. (b) Bar graphs showing MMP-1 protein levels analyzed by slot blot. Data are expressed as densitometric units and are mean ± SD for samples run in duplicate. (c) Bar graphs showing TIMP-1 gene expression after normalization on GAPDH mRNA levels. Data are expressed as mean ± SD for samples run in triplicate. ^∗^*p* < 0.05 and ^∗∗^*p* < 0.001.

**Figure 7:**
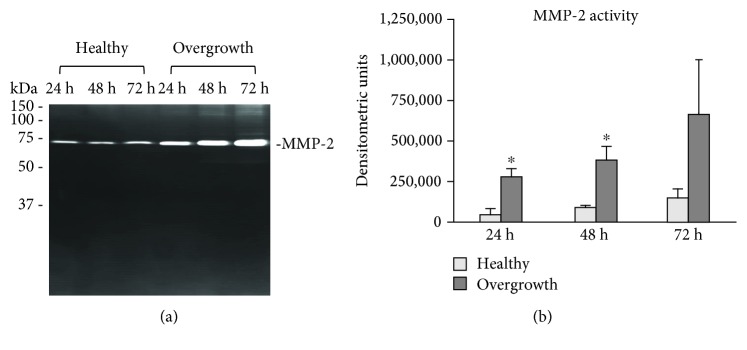
MMP-2 activity. (a) Representative SDS-zymography and (b) bar graphs showing MMP-2 activity in serum-free cell culture supernatants of fibroblasts from healthy and overgrown gingivae after densitometric analysis of immunoreactive and lytic bands. Data are expressed as densitometric units and are mean ± SD for samples run in duplicate. ^∗^*p* < 0.05.

Collagen degradation pathways were assayed by slot blot, real-time PCR, and SDS-zymography. MMP-1 levels tended to increase in fibroblasts obtained from the overgrown gingiva (*p* < 0, 05, *p* = 0, 06, and *p* = 0,073 vs. healthy gingiva after 24, 48, and 72 h, respectively) (Figures 6(a) and 6(b)). The expression of TIMP-1 mRNA, the main inhibitor of MMP-1, was unchanged after 24 and 48 h but strongly increased in fibroblasts from the overgrown gingiva after 72 h (*p* < 0,001) (Figure 6(c)). MMP-2 activity was characterized by a pattern concordant with MMP-1 protein levels (*p* < 0, 05, *p* < 0, 05, and *p* ns vs. healthy gingiva after 24, 48, and 72 h, respectively) (Figures 7(a) and 7(b)).

### 3.5. FAK Gene Expression

**Figure 8:**
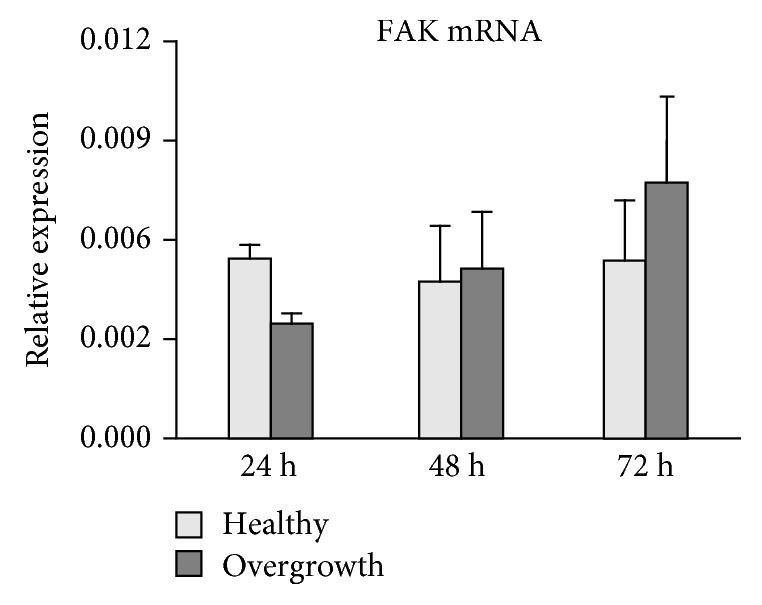
FAK mRNA levels. Bar graphs showing FAK mRNA levels after normalization on GAPDH gene expression. Data are expressed as mean ± SD for samples run in triplicate.

Gene expression analysis for FAK revealed similar mRNA levels in both experimental conditions after 24 and 48 h and a tendency towards upregulation in fibroblasts from the overgrown gingiva after 72 h (*p* = 0.066) (Figure 8).

## 4. Discussion

Alteration of the oral mucosa wounding affecting this patient suggests a possible relationship between keloid development and exostosis formation. To evaluate this association, we decided to analyze the healthy and overgrown gingivae overlying the exostosis of the same patient. According to the clinical observations, traumatism produced during the surgery seems to be relevant to the occurrence of relapses [1, 3].

Histological analysis revealed that the epithelium of the overgrown gingiva exhibited a normal general structure but showed an evident birefringence of the stratum corneum. We hypothesize that the mechanical stimulus exerted by the exostosis on the overlying gingiva resulted in a more compact stratum corneum, containing dense birefringent keratin bundles.

Collagen is the main component of the gingival dense irregular connective tissue. The histological analysis of the healthy and overgrown gingivae revealed a similar collagen content, as shown by Sirius Red staining. Polarized light microscopy revealed qualitative differences in collagen content, as the overgrown gingiva presented green-yellow coloration, while the healthy gingiva was stained in red, indicating that the collagen in the overgrown gingiva is less mature and therefore more susceptible to degradation by MMPs.

In order to understand the mechanisms responsible for this difference, we analyzed collagen turnover pathways in cultured gingival fibroblasts from the healthy and overgrown gingivae at the molecular level.

Collagen content is determined by the finely regulated dynamic balance between its synthesis and degradation by MMPs. MMP-1 begins collagen degradation breaking down the native triple helical region of interstitial collagens into characteristic 3/4- and 1/4-collagen degradation fragments, also known as gelatins [18], that can be further degraded to complete the digestion by less specific proteinases such as MMP-2 [19, 20]. MMP activity is regulated at the posttranslational level by TIMPs, inhibiting their activation and their activity [21]. Our results show that COL-I, the main component of gingival connective tissue, and COL-III protein levels were increased in supernatants of gingival fibroblasts from the overgrown gingiva. Moreover, an increased proliferation of fibroblasts obtained from the overgrown gingiva was observed. The apparent inconsistency between the morphological analysis, showing a similar collagen content, and increased collagen protein levels secreted by cultured fibroblasts obtained from the overgrown gingiva can be explained by analyzing the collagen degradation pathways. In fact, the observed concomitant increased MMP-1 and MMP-2 protein levels suggest an increased collagen turnover rate in fibroblasts from the overgrown gingiva. Consequently, increased collagen secretion in the overgrown gingiva is concomitant and paralleled with the increase in its degradation, resulting in a similar collagen content at the steady state in the connective tissue, as observed at the histological level. According to this suggestion, we can hypothesize that the observed TIMP-1 upregulation can be induced as a response to the MMP-1 overexpression, in order to balance it.

Newly synthesized collagen undergoes cross-linking, an important requirement for collagen maturation that provides collagen fibril stabilization and an increased tensile strength [22, 23]. Here, we show that LOX is strongly and significantly increased in fibroblasts from the overgrown gingiva, pointing to an increased collagen cross-linking activity induced by the exostosis.

The increased collagen turnover in fibroblasts from the overgrown gingiva could be therefore responsible for the differences revealed by the histological analysis. In fact, the exostosis could trigger a more rapid collagen turnover, consistent with the qualitative differences observed by polarized microscopy. However, in fibroblasts from the overgrown gingiva, LOX is significantly induced, suggesting that the newly secreted collagen in overgrown gingiva could undergo an increased cross-linking and maturation. Overexpression of LOX is very likely an adaptive response to avoid an excess collagen degradation in the gingiva, and it could favor collagen deposition in the gingival connective tissue covering the exostosis.

Collagen turnover rates can be affected by drugs as well as mechanical stimulation. Previous studies have shown that mechanical loading stimulates extracellular matrix (ECM) protein production by promoting the release of growth factors, such as TGF-*β*1, FGF, and PDGF [24]. Mechanical loading of cells has also been shown to modulate ECM turnover by regulating the expression and activity of MMPs [25–27]. Finally, mechanical loading interacts with growth factors and cytokines to regulate ECM homeostasis in various tissues [27–31]. We feel that our hypothesis of the role of exostosis as a triggering stimulus that influences gingival fibroblast activity is strengthened by the observations of gingival overgrowth exclusive to exostosis sites. Multiple exostoses were observed, each one covered by a gingival overgrowth lesion.

These suggestions are consistent with the tensegrity model described by Ingber, referring to a model of tensional integrity based on a network of structures that mechanically stabilize themselves using tensile prestress [32, 33]. According to this model, the whole structure is placed in a state of isometric tension that makes it strong, resilient, and immediately responsive to external mechanical stresses. The tensegrity model, referred to cells, led to the concept that mechanical forces would be sensed by cells using their cytoskeleton. In fact, a connection between cytoskeleton in the cell cytoplasm and the surrounding ECM is provided by the transmembrane proteins, integrins, working as mechanoreceptors, and finds its most useful application in understanding cellular mechanotransduction [34]. Integrins, mediating the attachment of cells to the ECM, are transmembrane proteins that provide a bridge through which forces can be transmitted between the inside and the outside of the cells. In fact, their extracellular domain binds to ECM components, whereas its cytoplasmic domain links various intracellular proteins interposed between actin filaments and integrin, forming focal adhesion complexes [35]. Mechanotransduction, the process that transforms mechanical stimuli into chemical signals, involves mechanosensory units integrated in the cell membrane that transduce mechanical signals into a biochemical response through force-dependent changes, important for regulation of global functions of the cell. Our data, showing a tendency to an increased gene expression of FAK, one of the components of the focal adhesions acting as a mechanoresponsive apparatus, is consistent with this hypothesis. In fact, an increase in focal adhesions as a response to mechanical stimulation elicited by the exostoses could be considered an adaptive response of gingival fibroblasts.

According to this model, we can consider gingival fibroblasts as cells using tensegrity architecture for their organization. This suggestion is supported by several studies that investigated the expression of ECM proteins in fibroblasts embedded in collagen gels under stretched and relaxed conditions, showing that the fibroblasts in the collagen adopted a synthetic phenotype characterized by their ability to synthesize matrix proteins and inhibit matrix degradation [36].

In this study, only one case of gingival overgrowth has been examined and we are aware that additional cases could advance and confirm our hypotheses; unfortunately, cases of multiple exostoses are very infrequent and this is the only case that came to our attention to date. Nevertheless, the association between exostoses and gingival overgrowth is strengthened by the presence of the overgrowths exclusively in the connective tissue covering each exostosis.

**Figure 9:**
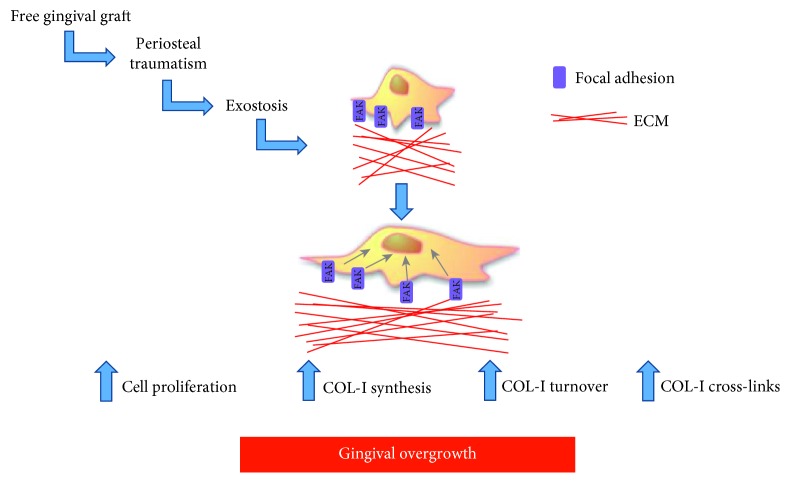
Diagram representing and summarizing the key events that could contribute to the development of gingival overgrowth covering the exostoses.

Our hypothesis is consistent with data previously reported [1, 3], showing a relationship between exostosis and FGG; these studies suggest that exostosis could be a rare side effect of FGG as a consequence of the periosteal traumatism during site preparation that induces the activation of osteoprogenitor cells. The clinical history of the patient revealed that the exostosis was formed after a surgical procedure that, very likely, triggered this mechanism inducing the exostosis, leading to the gingival overgrowth (Figure 9).

Our results suggest that exostosis could represent a mechanical stimulus stretching the overlying connective tissue and triggering a synthetic phenotype in gingival fibroblasts that, in turn, increase their activity in collagen turnover and maturation pathways. The effect of this stimulation seems responsible for the qualitative differences observed in the gingival connective tissue, thus affecting its biological and biomechanical properties. From a clinical point of view, the results of this study suggest that the surgical removal of such lesions, which is the only possible treatment, should be performed using minimally invasive surgical techniques to avoid traumatizing the periosteal tissues and triggering the mechanisms that can lead to gingival overgrowth. This procedure can be successful in the eradication of the exostosis with minimal relapses. A cautious use of drugs interfering with gingival collagen turnovers, such as cyclosporine A, anticonvulsants, and calcium antagonists is also advised.
